# Implementing virtual urgent care services in emergency departments: a multi-site focus group study of adaptation and sustainability

**DOI:** 10.1007/s43678-026-01109-2

**Published:** 2026-03-26

**Authors:** Mark Embrett, Sam Petrie, Sophia Salmaniw, Prosper Koto, Joanne Sanford, Janet Sommers, Tanya Penney

**Affiliations:** 1 Nova Scotia Health (Implementation Science Team), Halifax, NS Canada; 2 Nova Scotia Health (Virtual Urgent Nova Scotia), Halifax, NS Canada; 3 Department of Community Health and Epidemiology, Faculty of Medicine, Dalhousie University, Halifax, NS Canada

**Keywords:** Virtual care, Digital health, Implementation, Health policy, Provider perspectives, Soins virtuels, Santé numérique, Mise en œuvre, Politique de santé, Perspectives des fournisseurs

## Abstract

**Objectives:**

The Virtual Urgent Nova Scotia (VUNS) program was introduced to address emergency department (ED) overcrowding and improve access to urgent care through virtual health services. This study captures the perspectives, sentiment, and attitudes toward the implementation of VUNS from rural ED providers.

**Methods:**

VUNS staff from seven different sites across the province of Nova Scotia were invited to focus groups to explore the implementation of VUNS in their local context. Focus groups were facilitated by members of the Implementation Science Team at Nova Scotia Health from November 2024 to January 2025. Focus group transcripts were coded deductively, and results were presented in a narrative and descriptive fashion.

**Results:**

Key findings highlight recurrent issues, including staff confusion over inclusion and exclusion criteria, inconsistent VUNS physician decision-making, workflow disruptions influenced by staffing shortages, and patient bounce-backs from the VUNS triage nurses or physician. While initial implementation demonstrated potential for reducing lower acuity patients’ wait times and enhancing care delivery, operational challenges limited its effectiveness for some sites. Despite these barriers, success stories from specific sites emphasize the program’s value, particularly in underserved rural areas. Recommendations for improvement focus on stabilizing criteria, enhancing staff training, optimizing workflows, and fostering consistent communication between stakeholders.

**Conclusion:**

Findings indicate that VUNS should be scaled cautiously within current sites, addressing identified challenges before spreading to new locations. Lessons learned stress the importance of staff engagement, clear workflows, and patient education. These findings provide a roadmap for refining VUNS operations and contribute to the broader discourse on scaling digital healthcare innovations and streamlining patient flow in EDs.

**Supplementary Information:**

The online version contains supplementary material available at 10.1007/s43678-026-01109-2.

## Résumé

**Objectifs:**

Le programme Virtual Urgent Nova Scotia (VUNS) a été mis en place pour répondre à la surpopulation des services d'urgence (SU) et améliorer l'accès aux soins urgents grâce aux services de santé virtuels. Cette étude recueille les perspectives, les sentiments et les attitudes des prestataires de services d’urgence en milieu rural concernant la mise en oeuvre du programme VUNS.

**Méthodologie:**

Le personnel de VUNS provenant de sept sites diff érents de la province de la Nouvelle-Écosse a été invité àparticiper à des groupes de discussion afi n d’examiner la mise en oeuvre du programme dans leur contexte local. Ces groupes ont été animés par les membres de l'équipe de science de la mise en oeuvre de Santé Nouvelle-Écosse, entre novembre 2024 et janvier 2025. Les transcriptions ont été codées de manière déductive, et les résultats ont été présentés de façon narrative et descriptive.

**Résultats:**

Les principaux résultats mettent en lumière des problèmes récurrents tels que la confusion du personnel concernant les critères d’inclusion et d’exclusion, l’incohérence des décisions médicales des médecins VUNS, des perturbations dans les fl ux de travail causées par des pénuries de personnel, ainsi que le retour de certains patients vers les SU après évaluation par les infi rmières ou les médecins de triage de VUNS. Bien que la mise en oeuvre initiale ait montré un potentiel pour réduire les temps d’attente des patients à faible gravité et améliorer la prestation des soins, des défi s opérationnels ont limité son effi cacité dans certains sites. Malgré ces obstacles, des témoignages positifs issus de certains établissements soulignent la valeur du programme, notamment dans les zones rurales mal desservies. Les recommandations portent sur la stabilisation des critères, l’amélioration de la formation du personnel, l’optimisation des fl ux de travail, et la promotion d’une communication cohérente entre les parties prenantes.

**Conclusion:**

Les résultats suggèrent que l’expansion de VUNS doit être réalisée avec prudence dans les sites actuels, en s’attaquant aux défi s identifi és avant de s’étendre à d’autres lieux. Les leçons tirées soulignent l’importance de l’implication du personnel, de procédures claires, et de l’éducation des patients. Ces résultats off rent une feuille de route pour affi ner les opérations de VUNS et alimentent la réfl exion sur l’extension des innovations numériques en santé ainsi que sur l’optimisation de la gestion des fl ux de patients dans les services d’urgence.

## Introduction

Rural communities across Canada experience persistent inequities in access to health services compared with urban populations [1–3]. In Nova Scotia an Atlantic province on Canada’s East Coast, almost 40% of residents live in rural communities, and this population grew by 1.6% between 2016 and 2021—four times the national rural growth rate [4]. Despite this growth, many rural emergency departments (EDs) struggle to maintain physician coverage. In 2023–2024, for example, five EDs in Nova Scotia’s Western Zone experienced 15,769.5 h of temporary, unplanned closure, with similar disruptions in other zones [5]. Virtual care has been promoted as one way to improve access for rural patients, including through virtual urgent care [6–9]. Virtual urgent care has rapidly expanded across Canada, particularly in response to the COVID-19 pandemic when in-person services were sharply reduced, and evaluations showed only modest effects on downstream health services utilization, including subsequent ED visits following a virtual encounter [10].

To mitigate the impact of unplanned ED closures and support access to urgent care in rural NS, Virtual Urgent Nova Scotia (VUNS) was implemented across selected rural EDs and urgent treatment centers. This qualitative evaluation examined how the VUNS model functioned in practice within a rural and small-urban emergency care system, including perceived benefits, and unintended consequences from the perspective of staff responsible for delivering and supporting care. Using an implementation science lens, we sought to understand whether, how, and under what conditions VUNS should be sustained, modified, expanded, or discontinued within the provincial emergency care system.

## Methods

### Research design

This qualitative evaluation used a multiple-case study design [11, 12] to explore how the VUNS initiative was experienced and implemented across healthcare settings. This approach enabled an in-depth examination of contextual factors that shape the success of diverting non-urgent cases from EDs to virtual care. A comparative case study method [13] was employed to identify cross-site variations and thematic consistencies in implementation and outcomes. The focus groups concentrated on core domains of the Consolidated Framework for Implementation Research [14] and provided formative information on key considerations of the implementation of VUNS.

Data were collected through five semi-structured focus groups across seven sights, each lasting approximately 90 min in length, with healthcare providers and staff at seven VUNS sites between October 2024 and January 2025 (see Appendix A for the focus group guide). The focus groups’ evaluation questions focused on dimensions related to implementation, such as fidelity, engagement, leadership, communication, coordination, workflow, training, patient understanding, and experience [15], and were conducted in person (sites 1–4) or through a virtual platform (sites 5, 6, and 7) (Microsoft Teams). Supplementary documents, such as training materials, process maps, and analytic memos, were also reviewed to triangulate findings [16, 17].

Two researchers from Nova Scotia Health’s Implementation Science Team conducted the focus groups. The role of this team is to conduct independent evaluations of innovations across the health system. The researchers are PhD trained in evaluation, health policy & system research, and qualitative research.

### Case selection and participants

Each case represented an ED and its VUNS implementation. Sites were purposively selected [18] to reflect varied geographic regions, operational contexts, and implementation stages, with emphasis on early adopters. All selected sites were rural or remote. ED staff and providers (registered nurses, physicians, licensed practical nurses, clerical staff, and health services mangers) with VUNS experience were invited to participate. Demographic data were not collected due to the small size of the rural EDs and to protect anonymity. Data collection continued until thematic saturation was reached.

### Intervention

The VUNS pathway, launched in November 2023, allowed lower acuity emergency department patients (CTAS 3–5[19]) to be seen virtually by an emergency physician while physically present in a participating EDs. Emergency physicians were located in Ontario’s University Health Network and were approved by Nova Scotia Health medical affairs to provide virtual care in Nova Scotia. After triage and vital signs were completed by an on-site registered nurse, eligible patients with low-acuity presentations (e.g., cough, minor injuries, and follow-up of diagnostics) were offered VUNS and provided consent. Care was delivered via a tablet-based video visit with a VUNS emergency physician, while the ED nurse remained responsible for in-room assessment, repeat vital signs as needed, and implementing the care plan.

Virtual physicians could order laboratory and imaging tests through existing hospital systems (e.g., Meditech); where services were unavailable locally, patients were discharged with a referral and instructed to arrange testing in the community. If at any point the virtual physician or ED nurse felt an in-person assessment was required, the patient was seen face-to-face in the ED. VUNS typically operated from 08:00 to 20:00, with some variation by site. The technology platform was provided by Think Research, whose role was limited to queue management, eligibility workflows, and technical support; all clinical care was provided by emergency physicians in the centralized virtual care team. Initial inclusion/exclusion criteria were conservative and were iteratively revised by the provincial VUNS leadership and virtual care team, based on site feedback and experience, to broaden appropriate conditions while aiming to maintain safety and workflow feasibility.

### Thematic analysis

Thematic analysis [20] was used to identify patterns within and across cases. Each case was first analyzed independently by a member of the research team (SP and ME) to develop within-case themes. A cross-case analysis was then conducted and guided by the comparative case methodology [13]. The analysis was iterative and reflexive, involving deductive coding cycles and memo-writing. Discussion among the research team ensured analytic rigor and transparency. To ensure consistency, the team coded the first focus group transcript together, and the remaining transcripts separately.

### Ethics

This evaluation received a review exemption from the Nova Scotia Health Research Ethics Board (File #: 1,030,765). Participants were informed of project objectives prior to the focus group and provided verbal consent to be recorded. Consent was also implied through acceptance of the interview invitation and/or participation. Formal written consent was not required. All themes are reported in aggregate and anonymously.

## Results

The following findings are organized thematically across all participating sites and reflect key factors influencing the implementation of VUNS. Illustrative quotes are included to demonstrate how staff experienced the integration of VUNs into clinical workflows.

### Embedding virtual care into local systems: adapting to site-specific contexts

This theme draws on multiple implementation dimensions—particularly *workflow integration*, *staff engagement*, and *infrastructure*—to highlight how local context either facilitated or constrained successful integration. Although all sites faced the challenge of incorporating a centralized program into diverse local settings, the degree of local adaptation and flexibility was a distinguishing factor between high- and low-fidelity implementations.

Sites 1 and 6 demonstrated high implementation fidelity and serve as exemplars of successful contextual integration. Both sites benefitted from stable infrastructure and a flexible, empowered workforce. Site 1, for example, had a well-established workflow and a supportive leadership structure that eased the integration of VUNS:

“We’ve been able to make it work because we had support and already had the structure to add VUNS on top of what we do.”—Site 1.

Staff at Site 6 similarly reported infrastructure readiness, including continuous access to diagnostics, which facilitated smoother patient flow:

“We have lab and X-ray here all the time, so we just do it. That makes a huge difference.”—Site 6.

These sites also adopted a pragmatic approach to workflow integration, ensuring that virtual care could be layered into existing systems rather than displacing them. Both sites reported minimal patient ‘bounce-backs’ and described VUNS as easing the burden on regional centers, affirming its value:

“You know it’s a small amount [of patients] of what actually goes through… but it takes those visits away from the regional…”—Site 1.

In contrast, Sites 2 and 3 struggled with limited infrastructure, inconsistent workflows, and a lack of organizational readiness. At Site 2, the program had to be retrofitted into the existing system through staff improvisation:

“We were kind of just figuring it out as we went. Nothing about it matched what we were used to, so we had to change things to make it usable.”—Site 2.

Site 3 experienced a significant mismatch between the centralized VUNS design and local patient needs, staffing, and acuity patterns:

“It seems like this decision was made by people far from the bedside, without understanding what our patients need.”—Site 3.

These quotes illustrate a recurring issue: insufficient acknowledgement of local realities during initial program design. Staff at these sites faced operational hurdles due to unclear inclusion criteria and inconsistent onboarding.

Site 4 presented a mid-range implementation experience. Staff here recognized the potential value of VUNS but felt it required more localized adaptation:

“If we could expand the scope of VUNS, that would improve it greatly. You can have a Bluetooth stethoscope… that would expand a lot of what VUNS can do.”—Site 4.

They also advocated for a shift in scheduling to better align with patient flow:

“One improvement of VUNS could be improved upon is that we start earlier in the morning…”—Site 4.

Across other experiences (Sites 5, 6, 7), variation was seen in infrastructure, staffing models, and patient demographics. Site 7 initially lacked dedicated space and had poor Internet reliability, leading to implementation fatigue and inefficiencies:

“We weren’t designated to one room, so we had to keep moving our equipment… it was just chaos.”—Site 7.

With time, targeted changes—such as securing a stable room and improving internet access—led to significant improvements:

“It took me maybe two hours for one patient when we first started… Now, I register them in 5–10 min, and they’re seen much faster.”—Site 7.

However, staff repeatedly emphasized that without dedicated personnel, such as a float nurse, implementation placed undue strain on already stretched teams:

“It becomes an issue when they need something from nursing, but we already have critically ill patients in the department.”—Site 6.

“I’ve said it in multiple meetings… even if it was just an LPN to float, it would make a world of difference.”—Site 7.

This theme underscores that VUNS should not be deployed through a proscribed, top–down, standardized model.

### The complexity of inclusion and exclusion criteria: standardization in theory, ambiguity in practice

This section examines how VUNS inclusion and exclusion criteria shaped implementation fidelity, staff engagement, and workflow integration. Although criteria were intended to be communicated from the VUNS platform to sites, participants reported this rarely occurred. Across sites, staff described evolving and inconsistent criteria that sometimes-impeded appropriate care, with unclear protocols and strained local–virtual team dynamics compounding these challenges.

At Site 1, staff characterized the inclusion and exclusion criteria as a “necessary evil”—acknowledged as important, yet often limiting when applied in practice. Clinical teams emphasized the role of judgment and flexibility, particularly in cases that did not clearly fit existing categories. As one staff member explained:

“I don’t like exclusion inclusion, not a fan of it…if there’s a way for a patient to be seen… it’s about validating and it’s about plan direction.” (Site 1).

Site 1 approached criteria as a guide, allowing space for nuanced clinical assessment and patient-centered reasoning, especially when navigating ambiguous or ‘grey zone’ scenarios.

At Site 2, local staff frequently encountered conflicts between their own assessments of patient suitability and the decisions of VUNS physicians. This mismatch led to uncertainty and interruptions in workflow, as on-site staff found themselves obliged to defer to remote providers even when their perspectives differed.

“What I may feel is appropriate… the doctor may not feel it’s appropriate… sometimes… they were like no, because of this, this and this, I feel they need in person assessment.” (Site 2).

The absence of mechanisms for shared understanding or feedback meant that, in practice, patients were sometimes caught in the middle, and frontline staff had to balance their professional judgment with externally imposed decisions.

At Site 3, repeated changes to the inclusion and exclusion criteria led to widespread confusion and disengagement among staff. As the scope of eligibility narrowed, rejection rates increased and the perceived value of the VUNS program declined. One staff member described this shift:

“We had a dip in our numbers… The inclusion/exclusion criteria being changed multiple times led to a lot of lack of buy-in… Even when [the patient] fit that criteria, the VUNS doctors were bouncing them back.” (Site 3).

The frustration extended beyond shifting eligibility, as staff recounted instances where rigid application of the criteria failed to take clinical context into account.

“A patient had a tick bite and just needed treatment. But because his blood pressure was high, they wouldn’t see him—even though that was normal for him.” (Site 3).

For these providers, inconsistent and inflexible enforcement of the criteria contributed to a sense of futility and diminished confidence in the virtual care model.

Site 4’s experience was marked by inconsistency in the application of criteria and a lack of clear communication during rollout. Staff reported unpredictability in which patients would be accepted by VUNS physicians, leading them to rely more on their own judgment in the absence of reliable patterns.

“Sometimes inconsistent… They might see someone with [a sore throat] one day, not see someone [with a sore throat] the next day.” (Site 4).

This uncertainty was compounded by vague promises that clearer guidance would be forthcoming:

“We were told… in a month’s time we’ll have a better list of what the inclusion exclusion criteria was… so that was kind of odd.” (Site 4).

The lack of concrete, stable criteria made it difficult for staff to feel confident in their decision-making and undermined trust in the process.

A similar pattern was evident at Sites 5 and 7, where inconsistent or unclear guidance on criteria led to internal debate and a heightened sense of legal caution among staff. As one participant described,

“It became very muddy, and staff began questioning everything—are we legally allowed to do this?” (Site 5).

When the rules felt ambiguous or in flux, staff hesitated and often chose the path of least risk, resulting in missed opportunities for care and eroding confidence in leadership’s direction.

### Workflow disruption and the problem of bounce-backs: when virtual care loops back, the hidden costs of misalignment

A persistent challenge across most VUNS sites was patient “bounce-backs”—cases where patients were routed to the virtual stream, then deemed ineligible or needing further assessment, and returned to the main ED queue. These bounce-backs exposed problems with inclusion criteria, workflow integration, and perceived value. Although closely linked to how eligibility was interpreted and applied, their frequency made them a distinct implementation issue: instead of streamlining care, repeated transfers highlighted misalignment between virtual and in-person workflows, added delays, and eroded confidence in the model.

Site 1, despite issues with eligibility criteria, was a notable exception, with staff rarely reporting bounce-backs as a recurring problem. Staff attributed this to proactive documentation and careful pre-screening before referring patients to VUNS, drawing on their familiarity with both local patients and the virtual workflow to minimize returns after virtual assessment. In contrast, bounce-backs became a defining feature of early implementation at Site 2, frequently undermining staff morale and patient experience as discrepancies in clinical judgment between on-site staff and remote VUNS physicians led to patients being sent back to the ED queue. As one staff member described,

“It was causing more flip backs to the emergency department, which then…just delays the patient care.” (Site 2).

Rather than expediting service, the system introduced an additional layer of triage that prolonged wait times, requiring patients to be processed by two separate teams and compounding delays in treatment.

The impact of ‘bounce-backs’ was also pronounced at Site 3, where strict application of inclusion criteria and limited follow-through from virtual providers led to frequent patient returns to the ED. These repeated handoffs generated both emotional and practical burdens. As one staff member observed,

“At triage, people already have this kind of anxiety about wait times… They wait and give all their information, then get kicked back to the ED queue.” (Site 3).

The resulting uncertainty left both patients and staff frustrated, as triage teams could not reliably predict which cases would ultimately be accepted or rejected by VUNS, making workflow planning difficult and further lowering morale.

At Site 4, bounce-backs became a routine—and increasingly problematic—part of daily operations. The lack of clarity around VUNS eligibility meant that patients, after being initially engaged by the virtual stream, often felt abandoned when referred to the ED. As one staff member noted,

“We have days where we try to send 5 or 6 people and all those people get turned away… they’re like you’re just wasting my time.” (Site 4).

This pattern eroded patient trust and, over time, made staff reluctant to even suggest the virtual option, fearing it would be more trouble than it was worth for both parties.

A similar story played out across the multi-site environments of Sites 5, 6, and 7, particularly in settings where workflows remained unsettled. Here, bounce-backs frequently resulted in duplicated effort and increased administrative burden. Staff described feeling trapped between organizational directives and on-the-ground clinical realities, particularly when repeated assessments became necessary due to patients being cycled through both virtual and in-person streams.

“We had a couple that went in, and then the doc said no and sent them right back. We had to start from scratch.” (Site 5).

Not only did this waste clinical resources, but it also heightened liability concerns if documentation or coordination was lost during transitions, further straining already stretched ED teams and diminishing enthusiasm for the VUNS initiative.

### Leadership, communication, and staff engagement: leadership as a determinant of trust, morale, and buy-in

This section considers how leadership support, communication practices, and staff engagement shaped implementation fidelity, morale, and the overall perceived value of VUNS across sites. Leadership style, openness to feedback, and the consistency of communication set the tone for how teams navigated uncertainty, interpreted changes, and ultimately, whether they felt ownership over the new model or viewed it as an external imposition.

At Site 1, leadership was widely regarded as facilitative and present, actively fostering a learning environment where staff across disciplines were encouraged to communicate openly and support one another. Regular meetings for case review and quick responses to confusion were described as part of daily practice:

“We have really strong leadership. We meet regularly to review cases… if there’s confusion, we address it quickly.” (Site 1).

Informal, cross-disciplinary connections were valued, and VUNS team members were seen as proactive in maintaining patient-centeredness and accessibility. As one staff member noted,

“[The VUNS team are] very proactive at initiating… We can [text them]… We’re all for the patient… providing services [to the patient]” (Site 1).

This collaborative leadership approach enabled quick resolution of uncertainties, cultivated trust in the process, and empowered frontline staff to engage confidently and constructively with the virtual care model.

Site 2 offered a more mixed experience, where some local leadership support was present, but feedback mechanisms often felt non-substantive. Staff described a dynamic in which their input was acknowledged but not meaningfully incorporated into decisions, which were ultimately handed down from above. One team member described this sense of disconnect:

“I was giving my feedback… and they were just like, OK, but we’re still doing it. You know what I mean?” (Site 2).

The result was frustration and a perception that the value of frontline experience was minimized, undermining feelings of ownership and leaving some staff disengaged from the broader aims of VUNS.

Site 3 illustrated the consequences of a rigid, top–down approach to leadership. Here, staff recalled being told what to do without consultation or adequate preparation.

“We had a meeting. They said, ‘This is what you’re doing.’ No consultation.” (Site 3).

The lack of two-way communication or opportunities for input meant that adaptation to local realities was stifled, staff felt unprepared to handle challenges as they arose and buy-in was limited. Instead of building a sense of collective purpose, the implementation felt imposed and impersonal, leading to skepticism, inconsistent adoption, and diminished morale.

At Site 4, communication from leadership was present but inconsistent and often unclear. Staff described being on the receiving end of top–down decisions, with little space for site-level input or contextualization. As two staff members put it (with very similar wording to site 3),

“This is what we’re doing. No consultation.” (Site 4) and.

“This feels to me political, like a grasping at straws move.” (Site 4).

Changes to protocols and criteria were not always effectively communicated, forcing staff to interpret and adapt on the fly:

“The inclusion/exclusion criteria changed, but they didn’t tell us. We had to figure it out.” (Site 4).

This reactive approach left staff feeling unsupported, reliant on informal workarounds, and ultimately alienated from the program. The result was a passive withdrawal of confidence and engagement, as the burden of navigating uncertainty and incomplete information fell squarely on frontline teams.

Across the Sites 5, 6, and 7, the significance of leadership and communication became even more apparent. At Site 6, the presence of engaged, flexible management was frequently cited as a key to smoother implementation and higher staff morale. Here, staff described working collaboratively with leadership to anticipate and manage challenges:

“If I know it’s going to be a busy day, I bring in extra help—an extra clerk, or another staff member. We work together to make sure it runs smoothly.” (Site 6).

In contrast, Sites 5 and 7 struggled with leadership that communicated primarily through top–down directives, with little opportunity for input or discussion. As one staff member at Site 5 described,

“We had no presentation or education on what [VUNS] was really about. Just: ‘This is what you’re going to do now.’ It wasn’t good.” (Site 5).

These approaches led to confusion, resistance, and diminished trust in the VUNS initiative.

### Cross-site comparison

Table 1 summarizes multi-level factors shaping VUNS implementation across system, site, team, and patient domains. Successful sites (e.g., Sites 1 and 4) combined strong leadership engagement, operational readiness, and local flexibility, enabling smoother integration and higher staff engagement. At the site level, clear workflows and protected staffing supported adoption; at the team level, defined roles, interprofessional collaboration, and structured onboarding were key. Patient-level factors, such as consistent triage and appropriate referrals, influenced perceptions of effectiveness. Overall, system-wide emergency care innovations appear most successful when leadership, local adaptation, and frontline workflow realities are aligned.

**Table 1:** Cross-site comparison of VUNS implementation dimensions, multi-level factors that shaped VUNS implementation

Level	Dimension	Description
System level	Leadership and buy-in	Engagement from senior leadership and health system champions supporting VUNS implementation
	System readiness	Infrastructure, processes, and leadership capacity at the system level to support rapid deployment
	Standardization vs. local flexibility	Balance between centralized model design and site-level adaptation
Site level	Site-level leadership	ED management and departmental leaders driving local implementation and maintaining momentum
	Resource allocation	Availability of physical space, equipment, and protected staffing to support VUNS
	Integration into ED workflow	Embedding VUNS into triage or ED patient flow to minimize disruption
Team level	Role clarity	Staff understanding of their responsibilities in VUNS (navigator, triage, and virtual physician)
	Interprofessional collaboration	Effective communication and shared purpose across ED clinicians, navigators, and virtual providers
	Training and onboarding	Access to orientation or preparation materials for staff involved in VUNS
Patient level	Patient appropriateness and flow	Selection of appropriate patients for VUNS and managing bounce-backs or transfers to ED

## Discussion

### Interpretation of findings

Across sites, VUNS was experienced as both an enabler and a barrier. Where leadership was visible and inclusive, staff were engaged early. Consistent training and communication were aligned with a more fully integrated VUNS and perceived as useful for a small subset of low-acuity patients. In contrast, top–down decision-making, limited training, and frequent or poorly explained changes to eligibility criteria were associated with lower fidelity, “bounce backs” to in-person care, workflow interruptions, and frustration about discrepancies between on-site and virtual clinical opinions. Several participants questioned whether the benefits justified the additional nurse time and handoffs required, describing VUNS as something they had “made work” rather than an obviously value-adding service.

### Comparison to previous studies

Previous evaluations of virtual urgent and emergency care also highlight context-dependent impacts. In Ontario, ED-led virtual expansion during COVID-19 showed limited reductions in subsequent ED use and introduced operational challenges [10, 21]; staff in our study likewise described VUNS as sometimes duplicative, disruptive to flow, or of uncertain net benefit. An Australian Virtual ED model reduced presentations when tightly integrated with triage and local resources [22, 23], whereas uncertainty about inclusion criteria, staffing, and clinical responsibility often undermined integration in our sites. U.S. work on virtual observation units suggests that cost-effectiveness depends on home monitoring and local capacity [24], echoing concerns that virtual models without adequate staffing, diagnostics, and follow-up pathways may shift rather than resolve access pressures.

### Strengths and limitations

A key strength of this study is its multi-site, qualitative design, which enabled rich, contextualized insights into the implementation of virtual urgent care across diverse rural settings, including multiple professional roles also ensured a range of perspectives from frontline staff.

Limitations include participant demographics were not collected to preserve anonymity in small settings, limiting the ability to assess how experiences varied by role or tenure, because data relied on self-report and group discussion. There is also a risk of recall bias and social desirability bias. Finally, purposive sampling, while effective for capturing diverse perspectives based on participant characteristics, may limit the generalizability of findings due to potential selection bias.

### Health system implications

For health systems considering similar models, our results underscore that governance and implementation strategy are as important as the technology itself. Scale-ups should prioritize flexible implementation strategies, real-time feedback loops, and clearly defined inclusion criteria co-developed with frontline teams. Dedicated staffing, diagnostic access, and patient education will also be critical for scale-up, particularly in rural settings. Future virtual care initiatives should emphasize contextual fit, structural readiness, and staff empowerment.

### Implications for research

Future mixed-methods studies that include patient-reported experience and clinical outcomes, alongside staff perspectives, are needed to test which combinations of leadership behaviors, staffing models, and eligibility approaches most reliably yield sustainable and equitable outcomes.

## Conclusion

Virtual urgent care is a candidate model of care that aims to improve access to timely healthcare for rural and remote residents. This qualitative evaluation describes salient implementation elements which must be attended to, to facilitate a successful program in rural EDs. Having strong clinical guidance and support, top–down and bottom–up buy-in, and consistent communication are all important components to ensure that a virtual care program improves integrated care and does not fragment care further.

## Supplementary Information

Below is the link to the electronic supplementary material.

Supplementary file1 (DOCX 18 KB)

## Data Availability

The data that support the findings of this study are not publicly available due to confidentiality agreements with participants and the sensitive nature of the qualitative data. Participants did not provide consent for their full transcripts or identifying information to be shared beyond the research team. De-identified excerpts are available within the article to illustrate key themes.
