# Supplementary material for: Implementing virtual urgent care services in emergency departments: a multi-site focus group study of adaptation and sustainability
Source: CJEM. 2026 Mar 26;28(5):443–51. doi: 10.1007/s43678-026-01109-2 (PMC13161239; doi:10.1007/s43678-026-01109-2)
Supplement: Supplementary file 1 — Supplementary file1 (DOCX 18 KB) [file 43678_2026_1109_MOESM1_ESM.docx]

# APPENDIX A: Focus Group Guide – VUNS Implementation

### General Implementation Experience

Overall Experience:

1. How would you describe your overall experience with implementing VUNS?
   1. What went well?
   2. What challenges did you encounter?
2. Reflecting on the entire implementation process, what were the biggest successes?
3. What were the most significant challenges that you faced?
4. Thinking about your day to day, how had VUNS impacted your workflow?

Stakeholder Engagement:

1. From your perspective how did the collaboration of different stakeholders, such as leadership, staff, and patients, influence the implementation process?
   1. How may the collaboration be improved?
   2. How may challenges be addressed?
   3. Did you feel like you had an opportunity to provide feedback?
   4. If not, how would you have liked to provide feedback about the program?

### Fidelity to the VUNS Model

Adherence to Protocols:

1. From your perspectives to what extent did the VUNS implementation adhere to the planned model, protocols, and guidelines?
   1. Were there any elements that were difficult to implement as intended?
   2. Did you feel you had sufficient information/training about the program before it was implemented?
2. Were there any changes made to the approach during implementation?
   1. Why were these changes made, and what impact did they have on outcomes?
   2. Were these changes, and their reasoning, communicated clearly?
3. Can you share any examples where deviations from the approach that may have positively or negatively affected service delivery or patient outcomes?

### Factors Affecting Implementation

Enablers and Barriers:

1. What factors facilitated or hindered the successful implementation of VUNS?
   1. These could include resources, staff engagement, or support from leadership.
      1. PROBE: survey said:
         1. Allocating accommodation for consultation
         2. Consulting with VUNS provider
2. How did you address the barriers you encountered?
   1. Were there any strategies or solutions that worked particularly well?
3. How does existing organizational culture, policies, or leadership affect the implementation process?
4. Did the existing technological infrastructure or resource availability impact VUNS implementation? How?
5. Did staffing levels or staff training influence how VUNS was implemented?
6. Were resources (e.g., time, funding) adequate to support the program? Why or why not?
7. If you were to implement VUNS again, what changes would you make to improve adherence to the model and enhance outcomes?

### Overall Impact of VUNS

1. How has the implementation of VUNS influenced the overall delivery of services?
   1. Can you provide examples of specific improvements or challenges in day-to-day operations?
2. To what extent do you think VUNS has contributed to achieving broader organizational goals?
   1. Has it supported strategic objectives, such as improving patient care or lowering wait times?
3. In your opinion, what has been the most significant impact of VUNS? What aspects of the system do you see as the most valuable to patients, staff, and the organization?
